# Indulgent Parenting and Adolescents’ Maladjustments: The Roles of Cultural Context and Parental Gender

**DOI:** 10.3390/children11091132

**Published:** 2024-09-18

**Authors:** Qinglan Feng, Yanyun Yang, Ming Cui

**Affiliations:** 1 Department of Human Development and Family Science, Florida State University, Tallahassee, FL 32306, USA; 2 Department of Educational Psychology & Learning Systems, Florida State University, Tallahassee, FL 32306, USA

**Keywords:** adolescence, indulgent parenting, Bayesian SEM, maladjustments, cross-cultural

## Abstract

**Background/Objectives.** Adolescence is a critical developmental stage marked by the exploration of independence and self-identity. In this study, we aimed to examine the association between indulgent parenting (characterized by high responsiveness and low demandingness) and adolescents’ maladjustments across emotional, behavioral, and social domains. **Methods.** Using a cross-cultural sample of high school students from the U.S. (*n* = 268) and China (*n* = 189), we tested the hypotheses that indulgent parenting was associated with adolescents’ maladjustments, and that such association varied by cultural context (U.S. vs. China) and parental gender. **Results.** The results from Bayesian structural equation modeling supported the hypotheses, showing significant associations between indulgent parenting and adolescents’ maladjustments and differences in the associations across cultures and parental gender. **Conclusions.** The findings highlighted the need for culturally informed parenting programs to foster healthy adolescent development.

## 1. Introduction

Adolescence is a pivotal time for establishing crucial emotional, behavioral, and social habits that are foundational for mental health, with nurturing family environments being instrumental in this process [1]. At the same time, adolescence is a critical developmental stage characterized by seeking independence and self-recognition [2]. From the parenting perspective [3,4,5], indulgent parenting is characterized by a high level of warmth and a low level of behavioral control. As a multi-dimensional construct, indulgent parenting manifests in relational, behavioral, and material domains. It is conceptually similar to permissive parenting [3], but different from overparenting, helicopter parenting, or hovering in that indulgent parents are low in (behavioral) control rather than being over-controlling [6]. Indulgent parenting is an inappropriate parenting practice that conflicts with developmental needs during adolescence. The association between indulgent parenting and various aspects of adolescents’ maladjustments, such as emotional, behavioral, and social problems, has been documented with mixed findings. Furthermore, the contexts of these relationships remain unclear, with some studies suggesting that cultural context might play an important role. For instance, indulgent parenting has been generally associated with negative outcomes among U.S. adolescents (e.g., [7,8,9]), although with some exceptions ([10,11]). Some studies in other countries reported positive outcomes (e.g., [12,13,14,15]). Derived from the person–environment fit model [16] with cultural perspectives [17], adolescents are likely to adjust better in environments that resonate with their cultural attitudes, values, and experiences. This underscores the importance of considering cultural contexts in studies of parenting style. The role of indulgent parenting, however, is less explored in Eastern culture, with the outcomes mostly focusing on academic performance. The limited studies using samples from China also show mixed findings, with some suggesting parental indulgence related to better adjustments [18] and others suggesting it related to maladjustments among adolescents [19]. The inconsistent findings in the current literature indicate the potential significance of cultural differences in the effects of indulgent parenting; however, there is no cross-cultural study comparing the associations directly.

The role of parental gender in the context of indulgent parenting is also critical but underexplored. Although some research acknowledged the unique effects of maternal and paternal behaviors [19], much of the current knowledge is based on an overall assessment of parental indulgence, without differentiating between the roles of mothers and fathers (e.g., [12,20]). This approach may conceal important differences in how mothering and fathering separately contribute to adolescent development, potentially leading to an incomplete or biased understanding of parenting effects and family dynamics. This calls for more research into how maternal and paternal indulgent practices uniquely affect adolescent development.

Addressing these gaps, in the current study, we aim to deepen the investigation into the relationships between indulgent parenting and adolescents’ maladjustments in emotional (i.e., depression, anxiety, and stress), behavioral (i.e., aggression), and social domains (i.e., fear of missing out). We seek to elucidate the roles of cultural contexts by comparing the U.S. and China, and to explore the influence of parental gender, thereby providing a better understanding of the practices of indulgent parenting across different cultural contexts and parental gender.

### 1.1. Indulgent Parenting in Adolescence: Theoretical Perspectives

Indulgent parenting, combining high responsiveness with low demandingness, is one of the four parenting styles in the traditional parenting framework [4,5]. Parents who practice indulgent parenting are very responsive to their children’s needs and desires but do not demand responsible and mature behavior from their children. Indulgent parenting has been further operationalized into three dimensions: material, relational, and behavioral indulgence (e.g., [21,22]). Material indulgence refers to the ways in which parents might over-provide for their children’s material needs, such as buying children more toys, gadgets, and clothes than they need and not setting limits on spending. Relational indulgence involves the lack of boundaries and over-involvement in the parent–child relationship, such as being excessively involved in the child’s life choices and doing things that are supposed to be done by their children themselves. Behavioral indulgence focuses on the low expectations parents have for their children’s behavior, characterized by rarely enforcing rules through discipline and shielding their children from the consequences of their misbehaviors.

The parenting framework [4,5] has been substantiated through empirical research examining the relationship between parenting styles and child and adolescent development [23,24]. This body of research has indicated that children and adolescents raised with indulgent parenting exhibited less favorable developmental outcomes, such as lower levels of self-assertiveness and competence. Additionally, research has uncovered variations in these outcomes across different racial, ethnic, and gender groups. While Baumrind’s typology of parenting styles has been underpinned by some empirical research, the core of her work is largely theoretical in nature [25].

Consistent with established parenting theories, other developmental frameworks also acknowledge the association between indulgent parenting and adolescents’ maladjustments, suggesting that such parenting is a mismatch with adolescents’ developmental needs. The psychosocial development theory [2] marks adolescence as a critical period for forging a strong and individual sense of identity with clear boundaries from others, such as parents. Echoing Erikson, Havighurst [26] emphasized adolescence as a time to prepare for adult roles such as partnership and family life, to achieve emotional independence from parents, and to develop a value system to guide behavior. Likewise, self-determination theory highlights the burgeoning psychological needs of adolescents for autonomy, competence, and relatedness, advocating for parenting approaches that evolve to meet these needs [27]. Developmentally attuned parenting in adolescence entails fostering socialization in a way that promotes independence while reinforcing a stable sense of self with clear guidance to certain behaviors. Although indulgent parenting is permissive and supportive of self-expression, it frequently lacks the provision of essential boundaries and structure. While such parenting may foster openness and acceptance, allowing for freedom and self-exploration, it may inadvertently impede psychological growth by not sufficiently encouraging effort, responsibility, and the establishment of socially accepted values that direct behavior.

### 1.2. Indulgent Parenting and Adolescents’ Maladjustments

Indulgent parenting has not been as extensively researched as other parenting paradigms, although it has shown a growth trend in recent years [13,21]. Consistent with the theories, related research suggested an overall negative impact of indulgent parenting on adolescents across multiple domains including emotional, behavioral, and social outcomes [9,19,21,28,29]. The findings, however, are rather mixed, with some other studies suggesting the positive effects of indulgent parenting on adolescents’ growth, especially when considering cultural contexts (e.g., [11,12,30]). This inconsistency in findings warrants further investigation of cross-cultural differences in the relationship between indulgent parenting and adolescents’ maladjustments.

### 1.3. Indulgent Parenting in Cultural Contexts

Both the psychosocial development theory [2] and self-determination theory [27] suggest that indulgent parenting may not effectively meet developmental needs during adolescence. While extensive empirical evidence supports these theories, the presence of mixed findings indicates that these explanations may not be universally applicable. From an ecological perspective [31], the relationship between indulgent parenting and maladjustments could be more accurately understood within specific cultural contexts [17]. This perspective emphasizes that the cultural context, as part of the macrosystem, serves as the backdrop for all social interactions, including parenting. Parenting practices are deeply influenced by cultural norms and beliefs about what constitutes appropriate care and socialization of children or adolescents. Thus, culture not only shapes parents’ adaptation of parenting practices but also influences how adolescents perceive and react to their parents’ behaviors.

Research examining the relationship between indulgent parenting and adolescents’ maladjustments has been conducted across various countries, exploring a broad spectrum of emotional, behavioral, and social outcomes. These studies yielded mixed findings. For instance, indulgent parenting was linked to emotional maladjustments, such as heightened anxiety and stress, in a sample of American adolescents [8], while research with samples of European and Latin American adolescents found it associated with lower emotional irresponsiveness and instability in Spain [14], a lower level of anxiety in Germany [20], as well as a lower level of psychological dysfunction (e.g., in Mexico [15]). Mixed findings also emerged regarding the relationship between parental indulgence and adolescent behavioral maladjustments, such as aggressive behaviors, with some studies from Western cultures (e.g., Spain [32]) presenting different conclusions compared to those from Eastern cultures (e.g., China [19]). Such discrepancies have been examined and often attributed to cultural contexts [13,30,32]. For example, in Latin American culture, which emphasizes a strong family orientation [33], indulgent parenting may be more culturally aligned and thus associated with positive rather than negative outcomes among adolescents.

The U.S. and China are particularly relevant for cultural comparison along the spectrum of Collectivism–Individualism [34]. Heavily influenced by Confucian principles that emphasize familial hierarchy and parental authority, the collectivistic culture in China supports the idea of self as an integral part of a collective group. In contrast, the individualistic culture in the U.S. promotes autonomy and personal achievement, with a strong emphasis on individual independence and achievement within family dynamics. These foundational cultural differences could extend to parenting practices and their impacts on adolescents. Comparing the family dynamics of these two countries can provide valuable insights into how cultural contexts influence the relationship between indulgent parenting and adolescent development.

### 1.4. Parental Gender

The literature reveals a gap in understanding the distinct roles of maternal versus paternal parenting practices in adolescent growth. While a few studies acknowledged the distinct impacts of maternal and paternal parenting behaviors on adolescents’ adjustments, much of the research on parenting has concentrated on general parenting styles or specifically explored the impact of maternal factors (e.g., [8]), often overlooking the role of fathers. Treating maternal and paternal parenting as a homogeneous unit or interdependent styles does not make it possible to reveal important differences in how mothering and fathering separately contribute to adolescent maladjustments, potentially resulting in an incomplete or biased understanding of family dynamics. Based on the findings of the studies that explored maternal and paternal influences separately [13,19,29,35], further investigation in this area was encouraged and needed.

The limited number of studies in the current literature seems to conclude that the distinct roles of maternal and paternal indulgent parenting in adolescent maladjustments are somewhat unclear. Gender role/schema theory [36] provides a theoretical and universalistic view of gender expectations in child-rearing and parenting practices. In traditional heterosexual families, females are usually expected to be the primary parent who takes care of the children. When mothers are expected to be warm, nurturing, and more emotionally available, the role of fathers in parenting is more related to authority, control, and discipline. Due to the fact that indulgent parenting is characterized by a high level of warmth and a low level of control, which deviates more from fathers’ gender roles in parenting, paternal indulgence may be more likely to be related to adolescent maladjustments. With a growing trend toward shared parenting responsibilities, however, fathers’ role in parenting is less explored, especially when considering cultural contexts.

### 1.5. The Present Study

The current study aims to fill existing gaps in the literature by investigating the association between indulgent parenting and adolescents’ maladjustments in the U.S. and China. Drawing on prior theoretical and empirical research, we hypothesized that adolescents’ perception of indulgent parenting would be positively associated with various aspects of maladjustments, including emotional (e.g., depressive symptoms, anxiety, stress), behavioral (e.g., aggression), and social issues (e.g., fear of missing out) (H1). Furthermore, we anticipated that there would be cultural differences in these associations between the U.S. and China (H2). Finally, we proposed that the roles of maternal and paternal indulgence in adolescents’ maladjustments would differ (H3). Building on previous research that identified distinctive effects of the three dimensions of indulgent parenting [8], the unique contribution of each dimension was investigated in this study.

## 2. Methods

### 2.1. Sample and Procedures

This study used data from two projects that studied indulgent parenting and adolescent development. One sample was obtained from the U.S. (*n* = 268) and the other from China (*n* = 189). Adolescents in the U.S. were recruited from high schools in a southern city, and adolescents from China were recruited from the city of Shanghai. After obtaining parental consent and adolescent assent, adolescent participants from both sites completed an online survey to report their perceptions of their mother’s and father’s indulgent parenting and their own well-being adjustments, along with demographic information. The survey employed identical measurements for both samples, using a back-translation method for the Chinese version to ensure direct comparability of the results.

The adolescent participants in the U.S. had an average age of 15.36, with ages ranging from 12 to 18 years. Among these participants, 52.5% were African American, 38.6% White, and 8.9% from other races. A majority were female (55.2%) and non-Hispanic or Latino (85.2%). In the Chinese sample, the average age was 16.27, with an age range from 15 to 18 years. Approximately half were female (51.5%). Additional demographic details are provided in Table 1. This table illustrates similarities (e.g., gender distribution and family income) but also some slight differences between the U.S. and Chinese samples. Notably, the mean ages were similar, though the Chinese participants displayed less age variation. Furthermore, the Chinese sample had a higher percentage of participants from two-biological-parent family structures compared to the U.S. sample.

### 2.2. Measures

#### 2.2.1. Maternal and Paternal Indulgent Parenting in the U.S. and China

Indulgent parenting was assessed using a 30-item scale consisting of three dimensions: material, relational, and behavioral indulgence. Each item was measured on a 5-point Likert scale [22] (1 = Strongly disagree to 5 = Strongly agree), with maternal and paternal indulgence evaluated separately. Sample items included “My mother/father gives me all the clothes I want” (material indulgence); “My mother/father tries to make me dependent on her” (relational indulgence); and “When I break a rule outside of home, my mother would help me to avoid the consequences” (behavioral indulgence). For each dimension, items were summed after some items were reverse-coded, with higher scores indicating greater levels of indulgence. The scale demonstrated acceptable internal consistency reliability, with Cronbach’s alpha values ranging from 0.74 to 0.88 for maternal indulgence and from 0.73 to 0.93 for paternal indulgence in the U.S. sample. For the Chinese sample, the values ranged from 0.63 to 0.93 for maternal indulgence and from 0.71 to 0.94 for paternal indulgence.

#### 2.2.2. Adolescents’ Maladjustment in the U.S. and China

Adolescents’ maladjustments were measured through symptoms of depression, anxiety, stress, aggression, and fear of missing out (FoMO). Depressive symptoms were evaluated using the CES-D [37], which comprises 10 items, with a Cronbach’s alpha of 0.79 for the U.S. sample and 0.88 for the Chinese sample. Participants were asked how they felt regarding each statement during the past week on a 4-point Likert scale from “1 = Rarely or none of the time (Less than 1 day)” to “4 = Most or all of the time (5–7 days)”. A sample item is “I was bothered by things that usually don’t bother me.”

Anxiety was assessed with the BAI [38], consisting of 10 items, with alphas of 0.89 for the U.S. sample and 0.94 for the Chinese sample. Participants were asked to rate how much they have been bothered by that symptom listed during the past month on a 4-point scale from “1 = not at all” to “4 = severely-it bothered me a lot”. A sample symptom in the list is “Unable to relax.”

Stress was measured by the Rhode Island Stress and Coping Inventory [39], which includes 7 items, with alphas of 0.88 for the U.S. sample and 0.94 for the Chinese sample. Participants were asked to rate the frequency of how often each statement was true of their own life, using the 5-point scale (1 = Never to 5 = Frequently). A sample statement is “I felt there was not enough time to complete my daily tasks.”

Aggression was evaluated using a short version of the BPAQ [40], comprising 9 items, with alphas of 0.79 for the U.S. sample and 0.92 for the Chinese sample. Participants were asked to indicate how much each statement is like them based on a 5-point scale (1 = not at all to 5 = Exactly). A sample statement is “If someone hits me first, I let them have it.”

FoMO was assessed using Przybylski et al.’s scale [41], featuring 10 items, with alphas of 0.89 for the U.S. sample and 0.92 for the Chinese sample. A collection of statements about everyday experiences with peers and friends was presented to participants and they were asked to rate each statement based on a 5-point scale (1 = Not at all true of me to 5 = Extremely true of me). A sample statement is “I fear others have more rewarding experiences than me.”

### 2.3. Analytical Strategies

In this study, the key variables included adolescent reports of three dimensions of indulgent parenting—material, relational, and behavioral indulgence—and five domains of their maladjustments, namely depressive symptoms, anxiety, stress, aggression, and fear of missing out. Preliminary analyses involved descriptive statistics and correlations among these key variables.

#### 2.3.1. Structural Equation Modeling (SEM)

**Figure 1:**
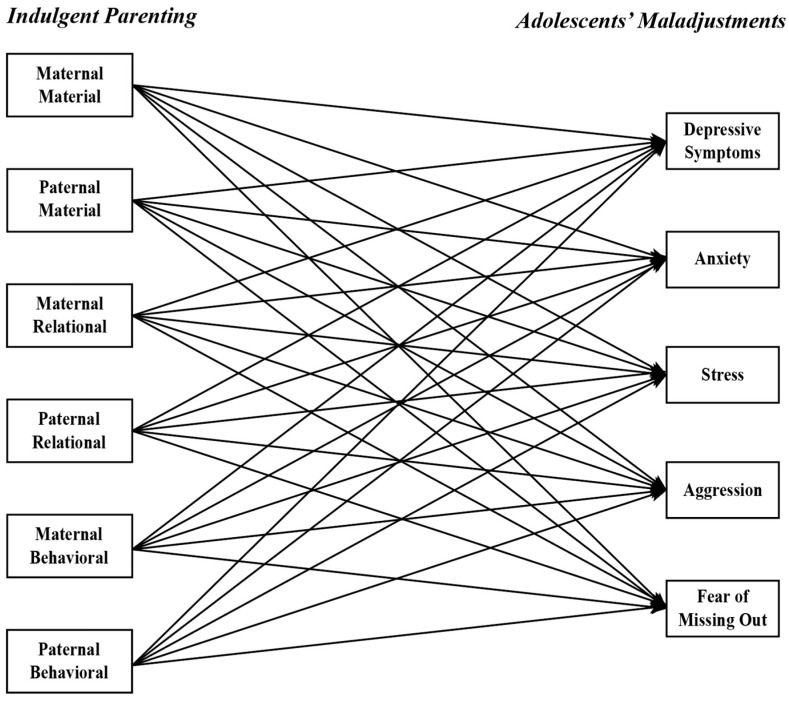
The proposed structural equation/path model. Exogenous variables are correlated with each other and the disturbances of endogenous variables are correlated with each other.

Path models in structural equation modeling (SEM) were utilized to examine the associations between indulgent parenting and adolescent maladjustments, as depicted in Figure 1. Specifically, maternal and paternal parenting practices were used as separate exogenous variables across the three dimensions of indulgence, resulting in six exogenous variables: maternal material indulgence, paternal material indulgence, maternal relational indulgence, paternal relational indulgence, maternal behavioral indulgence, and paternal behavioral indulgence. The five maladjustment variables were designated as endogenous variables. The model specification was the same for the U.S. sample and the Chinese sample. The model for the two samples was tested simultaneously by conducting a two-sample path model to allow for testing differences in associations across cultures. It was expected that, in each sample, the three dimensions of indulgent parenting would be positively related to the five domains of adolescents’ maladjustments (H1).

To test cultural differences (H2), comparisons were made between equivalent paths in the U.S. and Chinese samples. For example, to determine whether the path coefficient from maternal material indulgence to depressive symptoms was different across cultures, a parameter representing the difference in this path coefficient between the two samples was specified and tested for significance. A similar approach was used to test the hypothesis regarding parental gender differences (H3). A significant estimate of the difference parameter would indicate the association to be different across cultures or parental gender.

#### 2.3.2. Bayesian Structural Equation Models (BSEM)

This study utilizes a Bayesian approach to conduct the SEM analysis, marking a tentative and innovative shift from traditional frequentist methods commonly used in this research area. Given the complexity of the proposed models, the sample size of approximately 200 individuals per culture is considered relatively small. Frequentist approaches such as maximum likelihood (ML) estimation methods are prone to issues such as model non-convergence, inadmissible parameter solutions, and biased estimates in small samples within complex SEM analyses (e.g., [42]). In contrast, Bayesian structural equation modeling (BSEM) [43] has been recognized for its advantages in managing small sample sizes in psychological research (e.g., [44]), yet it remains underexplored in parenting studies. While not the primary focus of this research, the use of BSEM in this context may address methodological challenges and stimulate the broader application of Bayesian methods in future research.

The proposed two-sample model was conducted in M*plus* 8 [45]. In Bayesian analysis, a prior distribution should be specified for each model parameter (including the difference parameter). We used noninformative or diffuse priors implemented as default in M*plus* due to the limited and inconsistent findings from previous studies. The number of Markov chain Monte Carlo chains was two. The iteration number was 1000 with the first half of the iterations as burn-in. The model was determined to converge if the potential scale reduction factor was smaller than 1.05. The model and data yielded an adequate fit if the posterior predictive *p*-value for the difference between the observed and the replicated chi-square values was greater than 0.05 and smaller than 0.95 [43]. For each model parameter, the mean of posterior distribution was used as the point estimate. A parameter estimate was said to be significant if its 95% credible interval (C.I.) did not include zero.

## 3. Results

### 3.1. Preliminary Results

Table 1 provides demographic information for the samples from the U.S. and China. Table 2 details the descriptive statistics for the study variables. The results from the paired *t*-tests suggested significant mean-level differences between maternal and paternal indulgent parenting. Specifically, in the U.S. sample, maternal material and relational indulgence were higher than paternal material and relational indulgence, whereas paternal behavioral indulgence was higher than material behavioral indulgence. In the Chinese sample, paternal behavioral indulgence was higher than maternal behavioral indulgence. Preliminary correlational analyses are displayed in Table 3 and Table 4. Table 3 shows the correlations among the different dimensions of indulgent parenting of mothers and fathers in each sample. The correlations were mostly positive and significant, indicating relationships among different dimensions of indulgent parenting and between mothers and fathers in each sample.

Table 4 reports the correlations between indulgent parenting practices and adolescents’ maladjustments, which suggests some differences in the dimensions of indulgent parenting as well as across cultures. First, the correlations between behavioral indulgence and adolescents’ maladjustments were mostly positive and significant across both samples, providing preliminary support for H1. The correlations between material indulgence (mostly negative and significant) and relational indulgence (mostly non-significant) and adolescents’ maladjustments in the U.S. sample, however, suggested some contradictory findings to H1.

The correlations also provided some preliminary support for cultural differences (H2). Compared with those in the U.S. sample, the correlations between material and relational indulgence and adolescents’ maladjustments were positive in the Chinese sample. Furthermore, relational indulgence appeared to have stronger correlations with maladjustments among Chinese adolescents as compared to among American adolescents (e.g., r = 0.24, *p* < 0.01 in the Chinese sample and r = 0.05, not significant in the U.S. sample between parental relational indulgence and depressive symptoms).

Regarding parental gender differences (H3), the correlations between indulgence and adolescents’ maladjustments seemed to differ in all three dimensions of indulgence, though the patterns were opposite by culture. For example, the correlations between material indulgence and adolescents’ maladjustments suggested stronger negative associations among mothers than among fathers in the U.S. sample, but stronger positive associations among mothers in China. These preliminary findings suggested a more complex picture of the associations between different dimensions of indulgence and adolescents’ maladjustments by cultures and parental gender, guiding the subsequent BSEM analyses.

### 3.2. Hypotheses Testing

**Figure 2:**
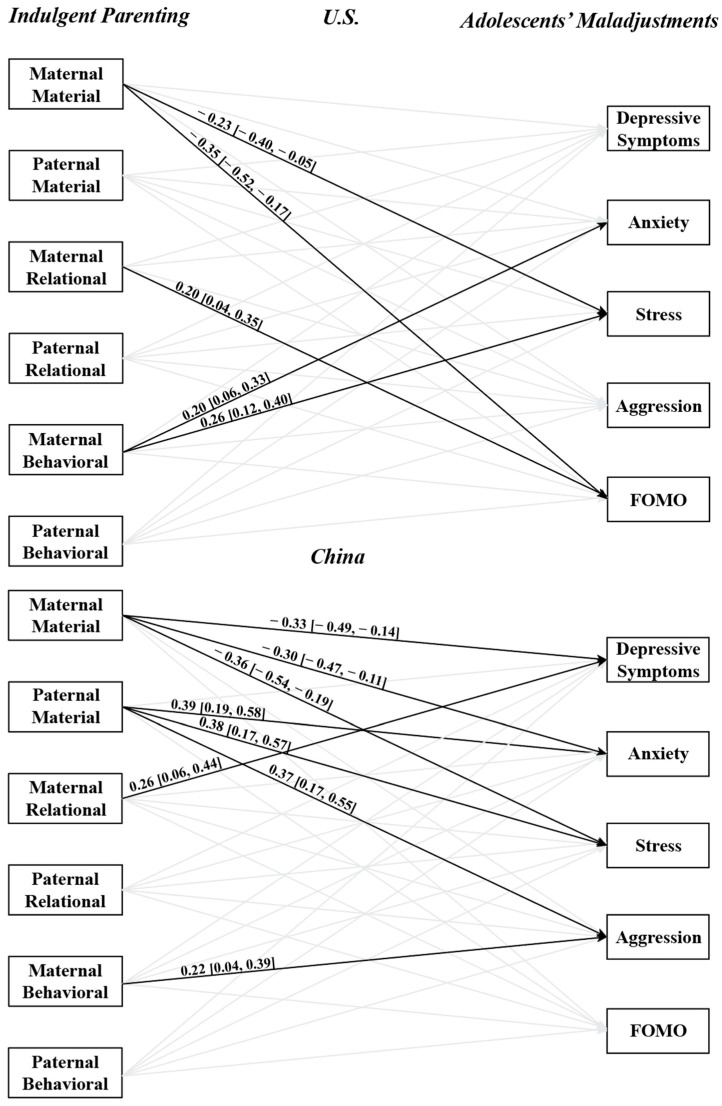
Bayesian SEM with key results: *n* = 268 for the U.S.; *n* = 189 for China. Bayesian 95% C.I. reported in []. Significant paths are shown with dark lines. Standardized estimates and 95% C.I. reported. All covariances between IVs were significant, except between maternal material indulgence and paternal behavioral indulgence in both cultures.

The three hypotheses were tested in the proposed two-sample model seen in Figure 1 using the Bayesian estimation method. The model converged, as indicated by the potential scale reduction factor of 1.005, smaller than 1.05. The model demonstrated an adequate fit to the data with a 95% confidence interval for the difference between the observed and the replicated chi-square values of (−12.18, 83.33), posterior predictive *p*-value = 0.098. The results are summarized in Figure 2. In Figure 2, all paths were tested but only significant path coefficients were illustrated. Table 5 presents the results of coefficient comparisons for cultural and parental gender differences. We now turn to each hypothesis testing in detail.

#### 3.2.1. Testing H1

The results for each sample are presented in Figure 2. In the U.S. sample, the path coefficients were significant only for maternal indulgent parenting. Specifically, maternal relational indulgence was positively and significantly related to adolescents’ FoMO (β = 0.20, 95%C.I. [0.04, 0.35], *p* < 0.05). Maternal behavioral indulgence showed positive and significant coefficients related to adolescents’ anxiety (β = 0.20, 95%C.I. [0.06, 0.33], *p* < 0.05) and stress (β = 0.26, 95%C.I. [0.12, 0.40], *p* < 0.05). Contrary to the hypothesis, maternal material indulgence was negatively and significantly related to adolescents’ stress (β = −.23, 95%C.I. [−0.40, −0.05], *p* < 0.05) and FoMO (β = −0.35, 95%C.I. [−0.52, −0.17], *p* < 0.05).

In the Chinese sample, both maternal and paternal indulgent parenting were related to adolescents’ maladjustments. As hypothesized, maternal relational indulgence was positively and significantly associated with adolescents’ depressive symptoms (β = 0.26, C.I. [0.06, 0.44], *p* < 0.05). Maternal behavioral indulgence was positively and significantly associated with adolescents’ aggression (β = 0.22, C.I. [0.04, 0.39], *p* < 0.05). The paths from material indulgence, however, diverged: paternal material indulgence was positively and significantly related to adolescents’ maladjustment including anxiety, stress, and aggression, while maternal material indulgence showed negative relations to depressive symptoms, anxiety, and stress. Taken together, the findings partially supported H1.

#### 3.2.2. Testing H2 and H3

Further statistical analyses were conducted to examine the difference in associations across cultures and parental genders, as detailed in Table 5. The findings revealed meaningful insights into cultural (H2) and parental gender (H3) differences in the associations between parental indulgence and adolescents’ maladjustments.

The analysis of cultural differences (H2) revealed significant variations in the relationships between parental indulgence and adolescents’ maladjustments across the U.S. and Chinese contexts. As illustrated in Table 5, a positive and significant coefficient indicates a stronger association in the Chinese sample, whereas a negative and significant coefficient suggests a stronger relationship in the U.S. sample. For material indulgence, regardless of parental gender, the path coefficients from material indulgence to adolescents’ maladjustments were generally stronger in the Chinese sample than in the U.S. sample. Specifically, the path coefficients from material indulgence to adolescents’ anxiety (95% C.I. [0.21, 0.65]), stress (95% C.I. [0.09, 0.51]), aggression (95% C.I. [0.09, 0.61]), and FoMO (95% C.I. [0.01, 0.55]) were significantly different between the U.S. and Chinese samples, with all paths being more pronounced in the Chinese sample. For relational indulgence, the paths from maternal indulgence to adolescents’ depressive symptoms (95% C.I. [0.02, 0.51]) and stress (95% C.I. [0.01, 0.56]) were stronger in the Chinese sample as compared to in the U.S. sample. The path from paternal relational indulgence to adolescents’ FoMO was also stronger in the Chinese sample (95% C.I. [0.05, 0.70]). Conversely, the path from maternal relational indulgence to adolescents’ FoMO was stronger in the U.S. sample than in the Chinese sample (95% C.I. [−0.71, −0.02]). For behavioral indulgence, the path from maternal indulgence to adolescents’ stress (95% C.I. [−0.73, −0.10]) was stronger in the U.S. sample than in the Chinese sample. These significant findings supported the cultural differences proposed in H2.

Regarding parental gender differences (H3), as detailed in Table 5, a positive and significant coefficient indicates a stronger relationship with maternal indulgence, while a negative coefficient suggests a stronger relationship with paternal indulgence. In the U.S. sample, notable differences were observed in the association of maternal and paternal indulgence to adolescents’ maladjustments. Specifically, paternal material indulgence had a stronger relation with FoMO than maternal material indulgence (95% C.I. [−0.79, −0.12]), whereas maternal relational and behavioral indulgence had stronger effects than paternal relational and behavioral indulgence on FoMO (95% C.I. [0.04, 0.76]) and stress (95% C.I. [0.09, 0.74]), respectively. In the Chinese sample, significant differences between maternal and paternal indulgence were found primarily in the associations with stronger paternal than maternal material indulgence to adolescents’ depressive symptoms, anxiety, stress, and aggression. These significant findings support the parental gender differences proposed in H3.

## 4. Discussion

Adolescence is a crucial developmental stage characterized by significant changes and challenges for adolescents. Central developmental tasks during this period include the pursuit of identity, independence, and autonomy, themes that are emphasized in various theoretical perspectives, such as the parenting framework [4,5], psychosocial development theory [2], developmental task theory [26], and self-determination theory [27]. Both theories and empirical evidence suggest that indulgent parenting—marked by high responsiveness but low demands—is linked to adolescents’ developmental maladjustments (e.g., [19,21,28,29]). These studies, however, often overlook how cultural contexts and parental gender could influence these associations.

Aiming to address the literature gap and further investigate the relationships between indulgent parenting and adolescents’ maladjustments, in the current study, we conducted Bayesian SEM analyses based on samples from the U.S. and China. Drawing on limited existing research, we hypothesized that adolescents’ perception of indulgent parenting would be positively associated with maladjustments (H1), with expected cross-cultural variations between the U.S. and China (H2), and differences in the effects of maternal and paternal indulgence (H3). The results generally supported our hypotheses.

### 4.1. Dimensional Indulgence and Adolescents’ Maladjustments

In general, the results of this study supported H1, according to which indulgent parenting is associated with adolescents’ maladjustments, including higher levels of depressive symptoms, anxiety, stress, aggression, and FoMO. Regarding behavioral indulgence, our findings suggested that behavioral indulgence demonstrated significant positive correlations with all aspects of adolescents’ maladjustments, indicating that higher levels of behavioral indulgence were associated with higher levels of depressive symptoms, anxiety, stress, aggression, and FoMO. This alignment with theoretical and empirical suggestions highlighted the significant risk that behavioral indulgence posed during adolescence [2,23,24]. In terms of parenting practices, paternal behaviors, such as setting rules but not enforcing them or shielding adolescents from the consequences of their actions, fail to meet adolescents’ need for clear boundaries. Such parenting behaviors undermine adolescents’ capacity to take responsibility and accept consequences, potentially contributing to maladjustments. This finding also echoes the notion that high levels of behavioral indulgence, in particular, could threaten adolescents’ psychological development [7].

Findings for material and relational indulgence, however, exhibited mixed results. In general, material indulgence was significantly associated with adolescents’ maladjustments in the current study but displayed both positive and negative directions in these associations. For relational indulgence, although some associations were negative, many were non-significant. These findings were consistent with some previous studies that also noted mixed directions in these associations, revealing the complex nature of indulgent parenting (e.g., [10,11,13]). These findings demonstrate that each dimension of indulgent parenting plays a unique role in these associations. This highlights the importance of examining indulgent parenting dimensionally rather than as a uniform concept. Furthermore, these mixed findings suggest the potential influence of cultural and parental gender differences, which we now turn to.

### 4.2. Cultural Differences between the U.S. and China

Taking a cultural ecological perspective [17,31], the findings from the current study provided support to the cultural differences hypothesized in H2 and indicated that the associations between indulgent parenting and adolescents’ maladjustments varied significantly across the U.S. and China. This study found that the positive associations between indulgent parenting and adolescents’ maladjustments were stronger in China, which suggested that the harmful effects of indulgent parenting on adolescents’ emotional, behavioral, and social development might be more salient in the Chinese culture. For instance, material indulgence was significantly and positively related to adolescents’ anxiety, stress, and aggression in the Chinese sample (but not in the U.S. sample). In addition, relational indulgence was positively related to adolescents’ depressive symptoms in the Chinese sample (but not in the U.S. sample). Although there were a few associations that were significant only in the U.S. sample, in general, the associations between indulgent parenting and adolescents’ maladjustments were found to be stronger in the Chinese sample than in the U.S. sample.

One possible explanation for the cultural differences found in this study is that indulgent parenting may deviate more from the traditional Chinese cultural norms. According to the stage–environment fit model [46], adolescents are more likely to experience adjustment difficulties in cultural environments that do not align with their attitudes, values, and experiences. Indulgent parenting (e.g., high warmth, low control) could contradict the Confucian values (e.g., parental authority and control) that are highly valued and prevalent in China [18]. Consequently, indulgent parenting may have stronger negative effects in China due to its poor alignment with cultural norms. With increasing globalization, however, the parenting trend is also changing in China, and more research is needed to further examine the nuanced cultural differences.

### 4.3. Gender Differences between Maternal and Paternal Indulgent Parenting

This study found that the associations between indulgent parenting and adolescents’ maladjustments varied significantly by parental gender, supporting H3. The results differed across the dimensions of indulgent parenting. For material indulgence, paternal indulgence was significantly and positively associated with adolescents’ aggression, whereas maternal indulgence’s association was not significant. This is consistent with the existing literature (e.g., [19]). Interestingly, while paternal indulgence was positively associated with adolescents’ anxiety and stress, maternal indulgence was negatively and significantly associated with these outcomes, suggesting that paternal material indulgence may play a detrimental role in adolescent development, whereas maternal material indulgence could be viewed as more beneficial. For relational and behavioral indulgence, maternal indulgence demonstrated stronger roles in adolescents’ maladjustments. Specifically, maternal relational indulgence was positively related to adolescents’ FoMO, and maternal behavioral indulgence was positively related to adolescents’ stress. These relationships were significant only for maternal indulgence and not for paternal indulgence.

Interestingly, cultural differences intertwine with gender differences. In the U.S. sample, only maternal indulgent parenting was significantly related to adolescents’ maladjustment, whereas in the Chinese sample, both mothers and fathers played a crucial role. This culture by parental gender difference may stem from cultural perceptions of the father’s role in parenting. In the U.S., a mother is typically viewed as the one more responsible for nurturing the child. In Chinese culture, rooted in Confucian values, there is a strong emphasis on the father’s role, as exemplified by the proverb “子不教, 父之过” (zĭ bù jiào, fù zhī guò), meaning “If the child is not taught, it is the father’s fault” (often attributed to Confucius).

Taken together, these findings underscore the necessity of separately investigating maternal and paternal parenting practices and considering parental gender differences in studies of indulgent parenting. The distinct impacts of maternal and paternal parenting on adolescents’ maladjustments, sometimes even contradictory, suggest that indulgent parenting can manifest as both positive and negative influences, depending on whether it is practiced by mothers or fathers. This distinction provides a valuable perspective for further exploring the conflicting findings in the current literature (e.g., [29,30]).

### 4.4. Application of Bayesian Structural Equation Modeling

The integration of Bayesian structural equation modeling (BSEM) into the current research offers a novel perspective within the field of parenting studies. BSEM is particularly advantageous for its ability to manage complex model structures alongside small sample sizes, which are prevalent in this field and present challenges for conventional frequentist methods, such as maximum likelihood estimation techniques [44]. By incorporating prior information, Bayesian methods enable a more flexible and nuanced inference, enhancing our confidence in interpreting the results from relatively small datasets. This study’s application of BSEM to examine the effects of indulgent parenting across diverse cultural backgrounds tentatively suggests the potential benefits of Bayesian approaches in this area. While the findings contribute to the ongoing dialogue in parenting research, they also invite further exploration and verification regarding the utility of Bayesian methods in this and related fields.

### 4.5. Limitations and Future Directions

Though filling a gap in the literature, this study has several limitations that warrant consideration. First, the samples collected from a single city or state are homogeneous to some degree, which may limit the generalizability of the findings to more diverse populations. Specifically, the Chinese sample was recruited in an urban setting with urban participants. Given the greater divide between urban and rural China in family demographics, the findings from the Chinese sample may not be generalized to the rural populations in China. Therefore, caution should be used in interpretations of the findings on cultural comparison. Second, the slight differences in distributions of age and family structure in the two samples may affect the direct comparability across these cultures. Third, this study did not examine covariates that might influence the relationships between indulgent parenting and adolescents’ maladjustments, such as adolescent gender, socioeconomic status, or educational background, suggested in previous studies [47]. Fourth, due to the cross-sectional design of this study, only correlation instead of causation was examined. The directionality of the effects observed cannot be definitively established. Lastly, while Bayesian methods offer a robust framework for analysis, model results may be sensitive to the specifications of prior distributions. In this study, due to the limited application of these methods in the existing literature, the priors were taken from the default settings in M*plus* 8 software. Relying on software defaults or diffuse priors, especially with small samples, may result in biased estimates, as suggested by McNeish [48].

Given the limitations and discoveries of the current study, future studies should aim to incorporate more diverse and comparable samples to enhance the generalizability of the findings across different populations. Including a range of covariates, such as socioeconomic status and educational background, could provide deeper insights into the complex relationships between indulgent parenting and adolescent outcomes. Finally, refining the specification of prior distributions in Bayesian analyses is crucial to ensure the robustness and accuracy of the findings, necessitating further research to validate and optimize these parameters.

## 5. Conclusions and Implications

This study contributed to the field by addressing existing gaps in the literature on indulgent parenting and its association with adolescents’ maladjustments across different cultural contexts. The findings highlighted the complex nature of parenting practices and their varied impacts on adolescent development, emphasizing the need for parents to adapt their approaches as their children grow and their developmental needs evolve. This study underlined the importance of considering both cultural differences and parental gender differences when designing and implementing parenting programs. Such programs should be tailored to reflect these variations to enhance their effectiveness. Furthermore, this study advocates for the development of culturally and gender-sensitive interventions that respect and incorporate the values and norms of the populations they aim to serve. For example, parent education programs can use culturally relevant scenarios in training materials to incorporate practical applications of parenting practices that align with the participating parents’ cultural values and experiences. Furthermore, these programs should include both mothers and fathers and discuss their distinct roles and expectations in parenting. Overall, by integrating these considerations, programs aimed at improving parenting practices can be more effectively aligned with the diverse needs of adolescents, potentially leading to better developmental outcomes.

## Figures and Tables

**Table 1:** Demographic information.

Demographic Information	U.S.	China
Sample Size	268	190
Mean of Age (Range)	15.36 (12–18)	16.27 (15–18)
Child Gender (Female)	55.2%	51.1%
Race (White/African American)	38.6%/52.5%	N/A
Ethnicity (Non-Hispanic or Latino)	85.2%	N/A
Family Income (below 150 k)	55.6%	54.7%
Family Structure (Two Biological Parents)	70.9%	90%

N/A = not applicable.

**Table 2:** Descriptive statistics on study variables.

Variables (*n*)	Descriptive Statistics			
	M (%)	S.D.	Min.	Max.
The United States (*n* = 268)				
Indulgent Parenting (Maternal/Paternal)				
Material Indulgence (*n* = 259/219)	**29.86/27.87**	7.70/9.67	10/10	50/50
Relational Indulgence (*n* = 259/219)	**27.71/25.30**	6.39/7.25	10/10	44/45
Behavioral Indulgence (*n* = 259/219)	**21.92/23.20**	5.54/5.59	11/12	37/35
Adolescents’ Maladjustments				
Depressive Symptoms (*n* = 240)	20.03	5.71	10	36
Anxiety (*n* = 240)	18.34	6.81	10	37
Stress (*n* = 238)	18.78	6.69	7	35
Aggression (*n* = 227)	22.03	7.24	9	45
Fear of Missing Out (*n* = 224)	22.77	8.79	10	50
China (*n* = 189)				
Indulgent Parenting (Maternal/Paternal)				
Material Indulgence (*n* = 179/172)	31.63/30.77	8.45/8.52	10/10	50/50
Relational Indulgence (*n* = 179/172)	26.13/26.55	6.71/7.69	10/10	46/46
Behavioral Indulgence (*n* = 179/172)	**27.78/28.85**	5.01/5.75	14/11	40/50
Adolescents’ Maladjustments				
Depressive Symptoms (*n* = 189)	20.20	6.59	10	40
Anxiety (*n* = 189)	18.25	7.55	10	40
Stress (*n* = 189)	16.96	7.27	7	35
Aggression (*n* = 189)	27.84	8.07	9	45
Fear of Missing Out (*n* = 189)	29.56	7.92	10	50

Note. Bolded pairs indicate significant differences by parental gender (*p* < 0.01 in paired *t*-test).

**Table 3:** Within-culture correlations among three dimensions of indulgence parenting.

		1	2	3	4	5	6
1.	Maternal Material	1.00	0.56 **	0.31 **	0.56 **	0.28 **	0.17 *
2.	Maternal Relational	0.51 **	1.00	0.32 **	0.44 **	0.60 **	0.31 **
3.	Maternal Behavioral	0.13 *	0.28 **	1.00	0.25 **	0.33 **	0.53 **
4.	Paternal Material	0.56 **	0.35 **	0.07	1.00	0.54 **	0.38 **
5.	Paternal Relational	0.29 **	0.48 **	0.23 **	0.59 **	1.00	0.46 **
6.	Paternal Behavioral	0.04	0.25 **	0.54 **	−0.01	0.21 **	1.00

Note. *n* = 268 for the U.S.; *n* = 189 for China. * *p* < 0.05, ** *p* < 0.01 (two-tailed). Lower triangle = U.S.; upper triangle = China.

**Table 4:** Within-culture correlations between indulgence and adolescents’ maladjustments.

Adolescents’ Maladjustments	Parental Indulgence: The U.S./China					
	Maternal Material	Maternal Relational	Maternal Behavioral	Paternal Material	Paternal Relational	Paternal Behavioral
Dep.	−0.18 **/−0.08	−0.04/0.19 *	0.17 */0.05	−0.09/0.16 *	0.05/0.24 **	0.19 **/0.22 **
Anxiety	−0.15 */−0.02	−0.01/0.15	0.20 **/0.05	−0.12/0.27 **	0.06/0.24 **	0.11/0.19 *
Stress	−0.23 **/−0.11	−0.12/0.08	0.22 **/0.01	−0.12/0.19 *	0.01/0.16 *	0.09/0.18 *
Aggression	−0.03/0.21 **	−0.02/0.13	0.05/0.30 **	−0.04/0.38 **	−0.04/0.23 **	−0.06/0.26 **
FoMO	−0.20 **/0.03	0.05/0.09	0.14 */0.06	−0.03/0.23 **	0.03/0.28 **	0.14 */0.13

Note. *n* = 268 for the U.S.; *n* = 189 for China. * *p* < 0.05, ** *p* < 0.01 (two-tailed). Dep. means depressive symptoms.

**Table 5:** Tests of cultural and parental gender difference in the association between indulgent parenting and adolescent maladjustments.

Difference	Estimate [95% C.I.]					
	Material		Relational		Behavioral	
Cultural	Mother	Father	Mother	Father	Mother	Father
Dep.	−0.14 [−0.33, 0.07]	0.18 [−0.02, 0.36]	0.27 * [0.02, 0.51]	−0.02 [−0.26, 0.20]	−0.20 [−0.48, 0.08]	0.08 [−0.18, 0.33]
Anxiety	−0.18 [−0.41, 0.04]	0.43 * [0.21, 0.65]	0.17 [−0.12, 0.46]	−0.11 [−0.38, 0.15]	−0.31 [−0.64, 0.01]	0.12 [−0.19, 0.44]
Stress	−0.13 [−0.35, 0.09]	0.31 * [0.09, 0.51]	0.27 * [0.01, 0.56]	−0.10 [−0.37, 0.15]	−0.40 * [−0.73, −0.10]	0.20 [−0.12, 0.50]
Aggression	0.02 [−0.24, 0.27]	0.36 * [0.09, 0.61]	−0.19 [−0.49, 0.11]	0.08 [−0.22, 0.37]	0.22 [−0.12, 0.57]	0.19 [−0.15, 0.52]
FoMO	0.28 * [0.01, 0.55]	0.09 [−0.18, 0.35]	−0.35 * [−0.71, −0.02]	0.37 * [0.05, 0.70]	−0.09 [−0.46, 0.32]	−0.22 [−0.58, 0.14]
Gender	U.S.	China	U.S.	China	U.S.	China
Dep.	−0.09 [−0.31, 0.14]	−0.41 * [−0.52, −0.16]	−0.06 [−0.33, 0.20]	0.24 [−0.09, 0.52]	0.08 [−0.21, 0.36]	−0.22 [−0.59, 0.14]
Anxiety	−0.01 [−0.25, 0.29]	−0.59 * [−0.90, −0.31]	−0.18 [−0.49, 0.12]	0.10 [−0.28, 0.47]	0.31 [−0.04, 0.64]	−0.12 [−0.56, 0.31]
Stress	−0.19 [−0.46, 0.07]	−0.63* [−0.92, −0.36]	−0.15 [−0.48, 0.15]	0.21 [−0.13, 0.56]	0.41 * [0.09, 0.74]	−0.19 [−0.60, 0.24]
Aggression	−0.01 [−0.32, 0.29]	−0.35 * [−0.68, −0.02]	0.08 [−0.25, 0.42]	−0.19 [−0.59, 0.20]	0.27 [−0.12, 0.64]	0.29 [−0.13, 0.72]
FoMO	−0.47 * [−0.79, −0.12]	−0.27 [−0.63, 0.08]	0.40 * [0.04, 0.76]	−0.32 [−0.74, 0.14]	−0.13 [−0.62, 0.31]	0.01 [−0.46, 0.51]

Note. *n* = 268 for the U.S.; *n* = 189 for China. Dep. means depressive symptoms. * indicates statistically significant differences with *p* < 0.05.

## Data Availability

Data used in this study are protected due to privacy.
